# Rapid On-Site Detection of SARS-CoV-2 Using RT-LAMP Assay with a Portable Low-Cost Device

**DOI:** 10.3390/bios13070724

**Published:** 2023-07-12

**Authors:** Quanying Fu, Xueyuan Pang, Zhenning Su, Yuxiao Yang, Yiren Liu, Ziyue Zhang, Yuqiu Fu, Jiasi Wang, Jianhua Zhou

**Affiliations:** 1 Guangdong Provincial Key Laboratory of Sensing Technology and Biomedical Instrument, School of Biomedical Engineering, Shenzhen Campus of Sun Yat-sen University, Shenzhen 518107, China; fuqy3@mail2.sysu.edu.cn (Q.F.); pangxy7@mail2.sysu.edu.cn (X.P.); yangyx79@mail.sysu.edu.cn (Y.Y.); liuyr33@mail2.sysu.edu.cn (Y.L.); zhangzy77@mail2.sysu.edu.cn (Z.Z.); fuyq7@mail2.sysu.edu.cn (Y.F.); 2 Guangdong Provincial Key Laboratory of Sensing Technology and Biomedical Instrument, School of Biomedical Engineering, Sun Yat-sen University, Guangzhou 510275, China; suzhn@mail2.sysu.edu.cn

**Keywords:** rapid on-site detection, portable low-cost device, nucleic acid detection, emerging infectious diseases, SARS-CoV-2 RNA

## Abstract

Emerging infectious diseases pose a serious threat to human health and affect social stability. In recent years, the epidemic situation of emerging infectious diseases is very serious; among these infectious diseases, severe acute respiratory syndrome coronavirus 2 (SARS-CoV-2) has affected many countries and regions in a short time. The prevention and treatment of these diseases require rapid on-site detection methods. However, the common detection method, RT-PCR, requires expensive instruments, complex operations, and professional operators. Here, we developed a portable low-cost assay for rapid on-site detection of viral nucleic acid using reverse transcription-loop-mediated isothermal amplification (RT-LAMP). The SARS-CoV-2 RNA can be successfully amplified within 15 min in a thermos, and the detection result is read rapidly in a portable low-cost device with a sensitivity of 10^0^ copies/µL. The portable low-cost device consists of a black box, a laser or LED and a filter, costing only a few cents. The rapid on-site detection method can provide strong support for the control of biological threats such as infectious diseases. It is also an emergency detection method for low-resource settings, relieving the huge pressure on health care.

## 1. Introduction

The continuous emergence of emerging infectious diseases has become an important factor threatening human health and affecting social stability. It is necessary to take urgent prevention and control measures against infectious diseases. In recent years, the representative emerging infectious disease is severe acute respiratory syndrome coronavirus 2 (SARS-CoV-2), which was discovered in 2019. It rapidly affected many countries and regions in a short time [1]. To control the spread of epidemic, early nucleic acid detection is an effective strategy [2]. The reverse transcription-polymerase chain reaction (RT-PCR) is presently gold standard in the detection of nucleic acids [3,4,5]. However, it requires specialized instruments and professional laboratories, which is not suitable for areas with limited medical resources [6]. Therefore, a rapid on-site detection method is still in great demand for the current complex medical environment.

Rapid on-site detection of nucleic acids requires simple amplification and detection methods to screen infected individuals as soon as possible [7]. The reverse transcription-loop-mediated isothermal amplification (RT-LAMP) assay can be used for rapid nucleic acid amplification [8]. It was used to detect SARS-CoV-2 in the early stages of the COVID-19 outbreak [9,10]. The great specificity, sensitivity and repeatability of the LAMP assay were also verified with the RT-PCR assay [11,12]. This assay can be carried out with a simple temperature control device, such as a thermos, which significantly improves the practicability and efficiency of rapid on-site detection [13,14]. The detection of nucleic acids methods combined with LAMP assays includes on-chip detection, naked-eye detection and cell phone imaging detection (Table S1). For on-chip detection, the isothermal amplification assay is helpful in the design of chips. Sun et al. used microfluidic chip amplification on a heating plate instead of the traditional RT-PCR method and mobile devices as detection instruments to detect pathogens, and the reaction were completed in 60 min. But the complex operation of chips made them more suitable for large-scale testing by health care workers [15]. For naked-eye detection, colorimetric indicators are usually used, but the sensitivity is lower than that of fluorescence detection. Lu et al. completed fluorescence monitoring within 30 min, while colorimetric detection required 40 min under the same conditions [12]. Fluorescence detection with higher sensitivity needs to be combined with image processing. For cell phone imaging detection, most photographs were taken on the devices. Chen et al. completed LAMP assays at 65 °C and used CRISPR/Cas2a at 37 °C for 40 min on a PCR instrument for detection. The results were observed by a portable 3D printing instrument and a cell phone [16]. Other commercial detection methods always rely on professional fluorescence detecting equipment, such as the HiberGene corporation detection instrument, which still has the problems of complexity and a high cost. These detection methods are often supplied for corporations or research organizations, rather than minimal medical resources. Therefore, in areas with limited medical resources, a portable device and detection method that can enter the home is needed.

**Figure 1:**
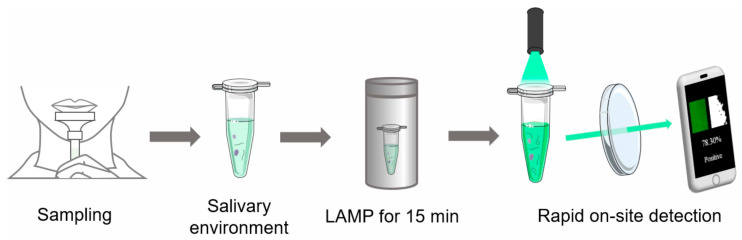
Schematic diagram of the rapid on-site detection method of SARS-CoV-2. Saliva was collected and added into a test tube with RT-LAMP premix, and the test tube was heated in a 65 °C thermos for 15 min; then, the photograph of test tube was captured and processed by a cell phone under a laser (LED also works).

Here, we developed a rapid on-site detection method of SARS-CoV-2 using a thermos and a portable low-cost device. The rapid amplification of nucleic acid with an RT-LAMP assay was completed in 15 min with a thermos (Figure 1). The on-site detection was completed in a portable low-cost device, which consisted of a black styrofoam box, a fixed light emitting diode (LED) or laser and a filter. The portable low-cost device only cost $0.83. Compared with the traditional RT-PCR and RT-LAMP assays, this detection method is rapid, low-cost and portable. We believe that this rapid on-site detection method will provide more possibilities for self-diagnosis in areas with limited medical resources and have extensive application prospects in on-site detection of emerging infectious diseases.

## 2. Materials and Methods

### 2.1. Reagents

Primers sequences were designed to be against the E, N and ORF3 gene of SARS-CoV-2 (NC_045512.2) using Primer Explorer V5 in Table S2. PCR and LAMP Primers and plasmids were synthesized by Sangon Biotech (Shanghai, China). EvaGreen 20× (25 µM) in water was purchased from Biotium, Inc. (Fremont, CA, USA). WarmStart^®^ LAMP Kit (DNA and RNA) was from New England Biolabs (Beijing, China). COVID-19 RNA reference material (high concentration, GBW(E)091089) was from the National Institute of Metrology of China. The RNA extraction kit was from Beijing Solarbio Science and Technology Co., Ltd. (Beijing, China). 

### 2.2. Salivary Environment

Saliva has been a promising sample that can be self-collected by potential patients, is convenient and decreases the risk to healthcare staff [17,18,19]. Saliva samples have also proved feasible for detecting SARS-CoV-2 [20]. Here, saliva was collected in a 5.0 mL test tube. The diluted saliva was mixed with ddH_2_O at a *v*/*v* ratio of 1:9. The 3 μL diluted saliva was taken by a calibrated quantitative capillary and blown into the reaction premix by mouth. After gently shaking and mixing the mixture, the reaction was carried out. The content of saliva had no effect on the qualitative results of this method within a 10-fold dilution (Figure S1). 

### 2.3. Primer Design

The primers for RT-LAMP and RT-PCR assays were produced and evaluated for hybrid structures by Standard Nucleotide BLAST (Table S2). The amplification of the ORF3 gene with primer-9 resulted in a false positive (Figure S2A). Primer-84 distinguished positives and negatives effectively (Figure S2B). Similarly, the primers for E and N genes were optimized. 

### 2.4. RT-LAMP Assay

The RT-LAMP assay took place in a thermos. The water in the thermos was a mixture of cold and hot water at 65 °C. A thermometer was used to monitor the water temperature. The RT-LAMP assay reagent comprised 12.5 μL of the WarmStart^®^ LAMP Kit (DNA and RNA), 1.6 μM inner primers, 0.4 μM loop primers, 0.2 μM bumper primers, 4× EvaGreen, 3 μL diluted saliva and a 1 μL sample. The LAMP Kit cost $1.06, the primers cost $0.046, the EvaGreen cost $0.014 and the reactants cost $1.12 per reaction.

### 2.5. Detection Device

After amplification, samples were placed in a portable low-cost device for photographing. The device consisted of a black styrofoam box, a 488 nm laser or a 485 nm LED array and a 520 nm filter. The laser or an LED array was secured in the black box and used to excite the fluorescence dye in the reagent. The light of laser was dispersed through a translucent cloth to form a uniform light. The sample was placed at a fixed position, and the light streamed in from the top of the tube. The photographs and extracted images were obtained by a cellphone with a filter on the window of the box. To analyze and process the images, a Python application was used which could read the brightness value of every pixel in the extracted image. All pixels with brightness value greater than the brightness threshold were adjusted to white, and all pixels with brightness value less than the brightness threshold were adjusted to black. Finally, the processed image and the percentage of white area was output; if the percentage of white area was greater than 0, the sample was considered positive, and the opposite was negative.

## 3. Results and Discussion

### 3.1. Optimization of RT-LAMP Assays and Determination of Brightness Threshold

**Figure 2:**
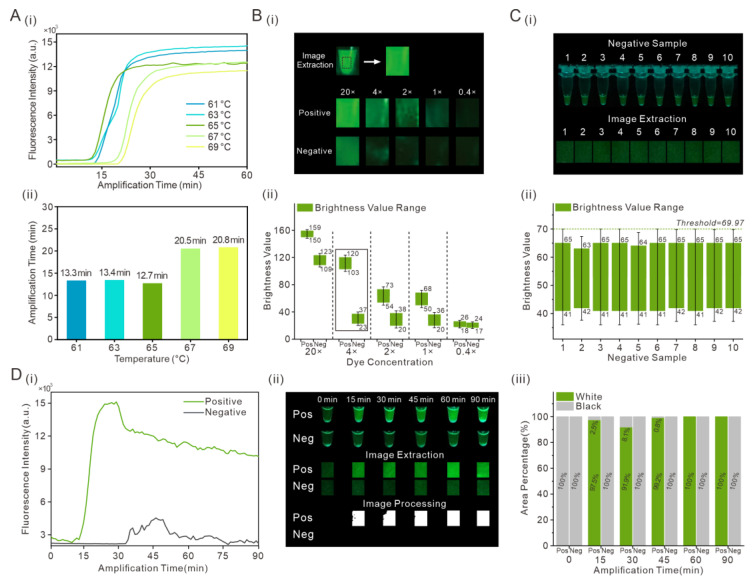
Optimization of the reaction parameters for RT-LAMP assay and determination of brightness threshold. (**A**) Effect of temperatures on RT-LAMP assay. (**i**) The amplification curves at 61, 63, 65, 67 and 69 °C; (**ii**) The reaction time at different temperatures. (**B**) Effect of different concentrations of dye. (**i**) Photographs of positive and negative samples with different concentrations of dye (20×, 4×, 2×, 1× and 0.4×); (**ii**) The Brightness value range of positive and negative samples with different dye concentrations. (**C**) Determination of the brightness threshold of the negative sample with dye at a 4× concentration. (**i**) Photographs of negative samples with dye at the 4× concentration; (**ii**) Brightness value range of negative samples. (**D**) The change of fluorescence intensity over time and image processing of positive and negative samples. (**i**) The amplification curves of positive and negative samples at 65 °C; (**ii**) Image processing of positive and negative samples from 0 min to 90 min; (**iii**) White and black area percentage of processed images.

To evaluate the feasibility of this detection method, the SARS-CoV-2 gene was used as the sample to be amplified in a thermos. The amplification parameters, reaction temperatures, dye concentrations and reaction time, were optimized (Figure 2). 

The amplification temperature is an important condition for the RT-LAMP assay, and the temperature in the thermos may change over time. The amplification curves of the RT-LAMP assay were obtained at 61, 63, 65, 67 and 69 °C in Figure 2A(i). The minimum amplification time was obtained when the amplification temperature was 65 °C (Figure 2A(ii)); the amplification time increased at other temperatures. The optimal reaction temperature was set to 65 °C in the thermos. The concentration of dye was selected, which has a significant effect on the method of image processing. The EvaGreen dye was altered according to the results of the amplification curves of different dyes in Figure S1C,D. As shown in Figure 2B(i), the photograph of the sample containing EvaGreen dye 20× was bright, and the contrast between the positive and negative samples was not obvious. The sample photographs were extracted and the brightness value was read in Figure 2B(ii). The range of difference of brightness values between positive and negative samples was only 27. The EvaGreen dye 20× (25 µM) in water was diluted to concentrations of 4× (5 µM), 2× (2.5 µM), 1× (1.25 µM), and 0.4× (0.5 µM) and the sample brightness value decreased. When the dye concentration was 4×, the range of brightness values between positive and negative samples was 66. When the dye concentration was 2×, the range of brightness values between positive and negative samples was 16; at a concentration of 1×, the range of brightness values between positive and negative samples was 14; at a concentration of 0.4×, the brightness value range overlapped. Therefore, when the dye concentration was 4×, the best detection results were obtained through image extraction and processing.

Under the conditions of an amplification temperature of 65 °C and a dye concentration of 4×, 10 negative samples were amplified, and the images were processed (Figure 2C(i)). The brightness values of the extracted images were read, respectively. The range of brightness values in the negative samples changed very little. The upper limit of error bar for the range of brightness values was 69.97, which was set as the threshold of image processing in Figure 2C(ii). After optimizing the amplification temperature and the concentration of the dye, the amplification process was sped up. In Figure 2D(i), the positive sample was amplified within 15 min. When the positive and negative samples were amplified in a thermos, the photographs were taken by cell phone. As shown in Figure 2D(ii), the image extraction was performed on the sample photographs. The extracted images were processed according to the threshold value obtained in Figure 2C(ii). All pixels with brightness values greater than the brightness threshold of 69.97 were adjusted to white, and all pixels with brightness value less than the brightness threshold 69.97 were adjusted to black. The percentage of white and black areas in processed image was shown in Figure 2D(iii). In the processed image of the positive sample, the percentage of white area was 97.5% at 15 min.

### 3.2. Evaluation of the Detection of SARS-CoV-2 in the Portable Low-Cost Device

**Figure 3:**
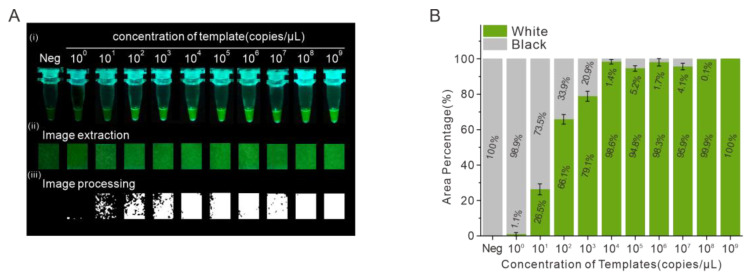
Evaluation of the sensitivity of the detection of the SARS-CoV-2 gene. (**A**) Image processing of samples with different template concentrations from 10^0^ to 10^9^ copies/μL. (**B**) White and black area percentage of processed images.

Under the optimized amplification conditions and image processing method, to determine the limit of detection (LOD) of the SARS-CoV-2 gene, the RNA reference material with 1.6476 × 10^10^ copies/μL was serially diluted with 10-fold serial concentrations from 0.988 × 10^9^ (~10^9^) to 0.988 × 10^0^ (~10^0^) copies/μL. The samples were amplified in the thermos for 15 min and then photographed in our portable low-cost device, finally images were extracted and processed by the Python application. As shown in Figure 3A, the images were extracted from the photographs, and the extracted images were processed to be white and black according to the brightness threshold of 69.97. The white area accounted for 1.1% of the whole image when the RNA concentration was 10^0^ copies/μL. With the increase of gene concentration, the percentage of white area of the image area increased. When the concentration of RNA was 10^1^ copies/μL, the percentage of white area was 26.5%; when the concentration of RNA was 10^2^ copies/μL, the percentage of white area was 66.1%; when the concentration of RNA was 10^3^ copies/μL, the percentage of white area was 79.1%. When the concentration of RNA was above 10^4^ copies/μL, the percentage of white area was above 90% (Figure 3B). The template solution was then serially diluted two-fold to 500 and 250 copies/mL. As shown in Table S3 and Figure S3A, ten replicates were tested for each concentration. Only two samples were detected as positive at 500 copies/mL. This determined the LOD as 10^0^ copies/μL. This rapid on-site detection was carried out in a salivary environment with the samples containing SARS-CoV-2 RNA templates, artificial saliva, real saliva and Helicobacter pylori, which represents the common bacteria in the oral cavity, Human Umbilical Vein Endothelial Cells (HUVEC), Human Keratinocyte (HaCaT) Cells and common virus influenza A and human papilloma virus were tested and proved that this detection method had good specificity for the detection of SARS-CoV-2 (Figure S4).

### 3.3. Optimization of the Portable Low-Cost Device

**Figure 4:**
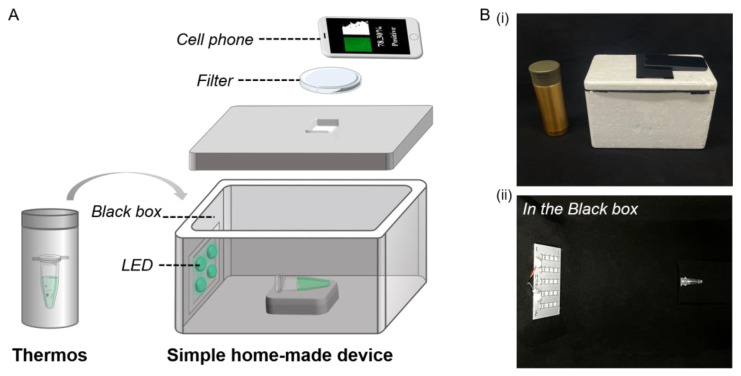
(**A**) Schematic of rapid on-site detection of SARS-CoV-2 gene by using the portable low-cost device. (**B**) Photographs of portable low-cost device and thermos (**i**) and the included tool in the device (**ii**).

Specifically, we improved amplification in a portable low-cost device. A thermos was used as the amplification device; the temperature of water in the thermos was adjusted by mixing hot water with cold water. The temperature of water could remain almost constant for 120 min (Figure S5). The portable low-cost device included a black box, an LED and a filter, as shown in Figure 4. After assembly, the sample was photographed by a cell phone under the excitation of a 488 nm laser or a 485 nm LED in Figure S6A. The image was extracted and processed by the Python application in Figure S6B,C. The light source of the LED was slightly less powerful than the laser. Therefore, the brightness threshold was adjusted to 66 under a 485 nm LED, and similar image processing results were obtained as under the 488 nm laser in Figure S6D. Both light sources can be used for detection; the LED is lower in price. 

The brightness values changed due to variation in the relative positions of the tube, excitation light source and detector. In Figure S7A(i), the relative positions of the tube and excitation light source changed to 5, 10 or 15 cm. When the distance between the tube and excitation light source was closer, the brightness value of the negative and positive were enhanced. As shown in Figure S7A(ii), when the distance was 10 cm, the difference of brightness values was larger. In Figure S7B, the relative positions of the tube and detector changed to 5, 10 or 15 cm. As the distance varied, the brightness value did not differ much. Therefore, we choose to read the brightness value range at a fixed position in the black box; the distance between the tube and excitation light source was 10 cm, and the distance between the tube and detector was 15 cm.

### 3.4. Detection of SARS-CoV-2 RNA Using the On-Site Detection Method 

**Figure 5:**
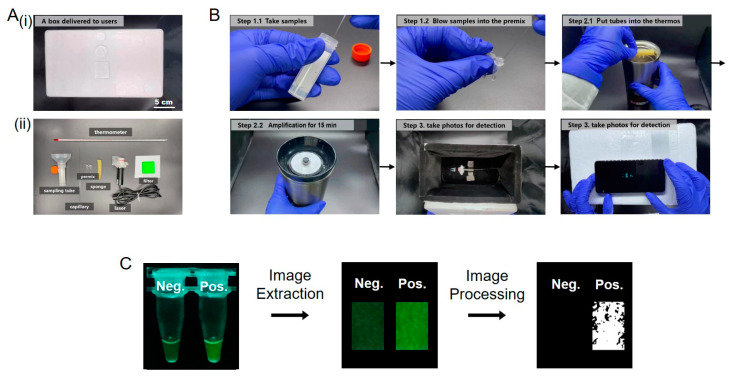
Photographs of portable low-cost device and the operation process of rapid on-site detection. (**A**) Photographs of portable low-cost device (**i**) and included tools (**ii**). (**B**) Operation process of rapid on-site detection. (**C**) The results of the RNA sample extracted from the SARS-CoV-2 transfected cells by using the portable low-cost device.

Finally, we built this portable device in Figure 5A. The device was low-cost and had a simple structure. The black box cost $0.27, the LED cost $0.42, the filter cost $0.14 and the entire detection device only cost $0.83. The thermos and cell phone were regular at-home equipment. As shown in Figure 5B and Video S1, in the practical application, users spit saliva into the water in the collecting tube; this was then quantitatively sampled using a capillary marked with a red scale. The saliva was blown into the reaction tube containing the premix (blow gently to avoid bubbles); then, the sample tube was put into the thermos for amplification. After 15 min of amplification, the tubes were positioned in front of the light source in the portable low-cost device; then, photographs were taken by cell phone, and images were extracted and processed by the Python application. Finally, users could observe the processed images and read the percentage of white area to judge whether the sample was positive or negative. The total time of the assay required about 22 min, as calculated in Table S4. RNA extracted from SARS-CoV-2 transfected cells in simulated samples was detected as in Figure 5C. 

In conclusion, some biosensors, such as antibody strips, are low-cost and user-friendly, but nucleic acid detection had higher clinical sensitivity and specificity [21]. RT-PCR is widely used for the screening of COVID-19 and other infectious diseases. The detection time usually requires 4–6 h, and the cost of a single kit may exceed $100 [22,23]. Even though some methods replace the fluorescence indicators to reduce the cost of the kit, there is still the problem of expensive equipment [24]. Some isothermal nucleic acid detection combined with CRISPR technology has a sensitivity of 6.75 copies/uL, but the detection time is usually 40–60 min [25,26]. Isothermal assays combined with colorimetric indicators, such as the colorimetric detection by Huang et al., have a sensitivity of 80 copies/mL, but the whole detection process usually takes 40 to 65 min [27]. Compared with these RT-PCR and LAMP methods, our detection method combines the advantages of these molecular diagnostic methods with fast detection speed, high sensitivity and low cost. The portable low-cost devices only cost a few cents and the reactants cost about $1.0 per reaction; this can be applied to rapid on-site detection in low-resource settings. 

## 4. Conclusions

We proposed a method for rapid on-site detection of SARS-CoV-2 using a portable low-cost device. An RT-LAMP assay was used for amplification in a thermos. The thermos is a common device in the home to provide more convenient conditions. The SARS-CoV-2 RNA was detected with a sensitivity of 10^0^ copies/μL in 15 min. The device was characterized by being inexpensive, with the value of the device not exceeding $1.0, and the total value containing the reagent not exceeding $2.0. The detection speed was rapid, and the total time of amplification and detection was less than 22 min. In summary, we developed a simple method to speed up the detection process, which has the benefits of simple equipment, low cost and simplicity of operation. The device can also be made smaller to make it easier to carry out portable inspections on site. In order to detect more infectious diseases, such as COVID-19, different excitation light sources combined with the filter can be used to perform multi-indicator detection of more pathogens. Our rapid on-site detection method and portable low-cost device could provide effective detection platforms for areas lacking medical resources.

## Data Availability

Not applicable.

## Supplementary Materials

The following supporting information can be downloaded at: https://www.mdpi.com/article/10.3390/bios13070724/s1, Table S1: The comparison and difference between our method and other portable LAMP methods; Table S2: Reference primers were used in the study; Table S3: Sensitivity of the LAMP assay for SARS-CoV-2; Table S4: The total time of the assay- from sample obtaining to results registration; Figure S1: Effects of saliva on the detection of RNA, Figure S2: The selection of primers and dye, Figure S3: Amplification curves and image processing of samples with different template concentrations, Figure S4: The specificity for detection of SARS-CoV-2, Figure S5: The temperature of water in the thermos, Figure S6: Effect of the excitation of LED or laser; Figure S7: The brightness value of sample changed with the relative positions of the tube, excitation light source, and detector; Video S1: Using process of the portable low-cost device. [12,15,16].
