# Supplementary material for: Rapid On-Site Detection of SARS-CoV-2 Using RT-LAMP Assay with a Portable Low-Cost Device
Source: Biosensors (Basel). 2023 Jul 12;13(7):724. doi: 10.3390/bios13070724 (PMC10377351; doi:10.3390/bios13070724)
Supplement: Supplementary file 1 [file biosensors-13-00724-s001.zip › biosensors-2470595-sup.pdf]

biosensors

Supplementary Materials

Rapid on-site detection of SARS-CoV-2 using RT-LAMP assay with a portable low-cost device

Quanying Fu[1,2], Xueyuan Pang[1,2], Zhenning Su[2], Yuxiao Yang[1,2], Yiren Liu[1,2], Ziyue Zhang[1], Yuqiu Fu[1], Jiasi Wang[1,2]* and Jianhua Zhou[1,2]*

1 Guangdong Provincial Key Laboratory of Sensor Technology and Biomedical Instrument, School of Biomedical Engineering, Shenzhen Campus of Sun Yat-sen University, Shenzhen, 518107, China.

2 Guangdong Provincial Key Laboratory of Sensor Technology and Biomedical Instrument, School of Biomedical Engineering, School of Biomedical Engineering, Sun Yat-sen University, Guangzhou 510275, China.*

Correspondence: wangjs8@mail.sysu.edu.cn (J. W.); zhoujh33@mail.sysu.edu.cn (J. Z.)

Table S1: The comparison and difference between our method and other portable LAMP methods in amplification, detection methodology and sensitivity.

	**Our method**	**Portable LAMP method**			**Commercially LAMP method**
		**Detection on chip**	**Detection by naked eyes**	**Detection with cellphone**	
**Amplification technique**	RT-LAMP	RT-LAMP	RT-LAMP	RT-LAMP	RT-LAMP
**Amplification and detection methodology**	Amplification with thermos, detection with a portable low-cost device.<br>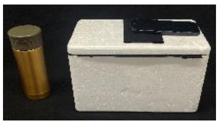	Amplification with heating plate, detection with manual separation.<br>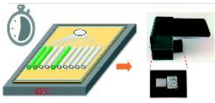	Amplification with thermostat water bath, detection by naked eyes.<br>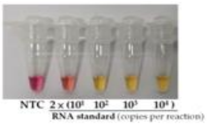	Amplification with the instrument, detection with 3D printing technology.<br>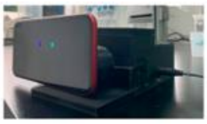	Amplification and detection with portable diagnostic instrument, the HG Swift.<br>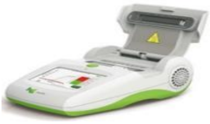
**Sensitivity**	High (25 copies per reaction)	High (18 copies per reaction)	Lower (200 copies per reaction)	High (20 copies per reaction)	-
**Total time**	~22 min	~60 min	~40 min	~40 min	30~60 min
**Reference**		*Sun et al, Lab Chip, 2020, 20, 1621* (ref.15)	*Lu et al, Int J Mol Sci, 2020, 21(8), 2826* (ref.12)	*Chen et al, Biosens Bioelectron, 2020, 169, 112642* (ref.16)	*HiberGene Corporation*

Table S2: Sequences of primers for loop-mediated isothermal amplification (LAMP).

LAMP primer	Sequence
**N gene-F3**	AACACAAGCTTTCGGCAG
**N gene-B3**	GAAATTTGGATCTTTGTCATCC
**N gene-FIP**	CGCATTGGCATGGAAGTCACTTTGATGGCACCTGTGTAG
**N gene-BIP**	TGCGGCCAATGTTTGTAATCAGCCAAGGAAATTTTGGGGAC
**N gene-LF**	TTCCTTGTCTGATTAGTTC
**N gene-LB**	ACCTTCGGGAACGTGGTT
**E gene-F3**	CCGACGACGACTACTAGC
**E gene-B3**	AGAGTAAACGTAAAAAGAAGGTT
**E gene-FIP**	CTAGCCATCCTTACTGCGCTACTCACGTTAACAATATTGCA
**E gene-BIP**	ACCTGTCTCTTCCGAAACGAATTTGTAAGCACAAGCTGATG
**E gene-LF**	TCGATTGTGTGCGTACTGC
**E gene-LB**	TGAGTACATAAGTTCGTAC
**ORF3-84-F3**	GCTTCCAAAATCATAACCCTC
**ORF3-84-B3**	GCAAAGCCAAAGCCTCAT
**ORF3-84-FIP**	ACAAACAACAACAGCAAGTTGCAATTTTAAAAAGAGATGGCAACTAGCA
**ORF3-84-BIP**	CTGGCCTTGAAGCCCCTTTTTTTTTTTATACTCTGCAAGAAGTAGAC
**ORF3-84-LF**	GAGGTTCCCACAAGTGAAAC
**ORF3-84-LB**	TCTATCTTTATGCTTTA
**ORF3-9-F3**	CACAATTGGAACTGTAACTTTG
**ORF3-9-B3**	GCCATCTCTTTTTGAGGGTTA
**ORF3-9-FIP**	TATCGTTGCAGTAGCGCGAATTTTAAGCAAGGTGAAATCAAGGA
**ORF3-9-BIP**	CCGATACAAGCCTCACTCCCTTTTATTTTGGAAGCGCTCTGA
**ORF3-9-LF**	CGATGAGGAAGTCTAAA
**ORF3-9-LB**	GATGGCTTATTGTTGGCGTT
**ORF3-42-F3**	ATTTTGTTCGCGCTACTG
**ORF3-42-B3**	CAAAAGGTGTGAGTAAACTGT
**ORF3-42-FIP**	CTCTGAAAAACAGCAAGAAGTGCAATTTTACGATACCGATACAAGCC
**ORF3-42-BIP**	CATAACCCTCAAAAAGAGATGGCATTTTCAAACAACAACAGCAAGTTG
**ORF3-42-LF**	GAGGGAAAGCCTACCGAATA
**ORF3-42-LB**	TAGCACTCTCCAAGGGTGTT
**PCR-1F**	CACTCCCTTTCGGATGGCTT
**PCR-1R**	TGAACACCCTTGGAGAGTGC

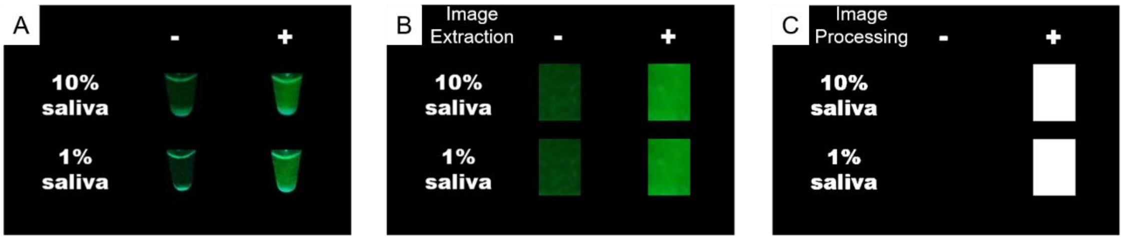

Figure S1: Effects of different amounts of saliva on the detection of RNA. A) Photos of samples containing 10% and 1% v/v saliva after 60 min amplification. B) Extracted images of samples containing 10% and 1% v/v saliva after 60 min amplification. C) Processed images of samples containing 10% and 1% v/v saliva after 60 min amplification; white area in the processed image of positive samples is 100%.

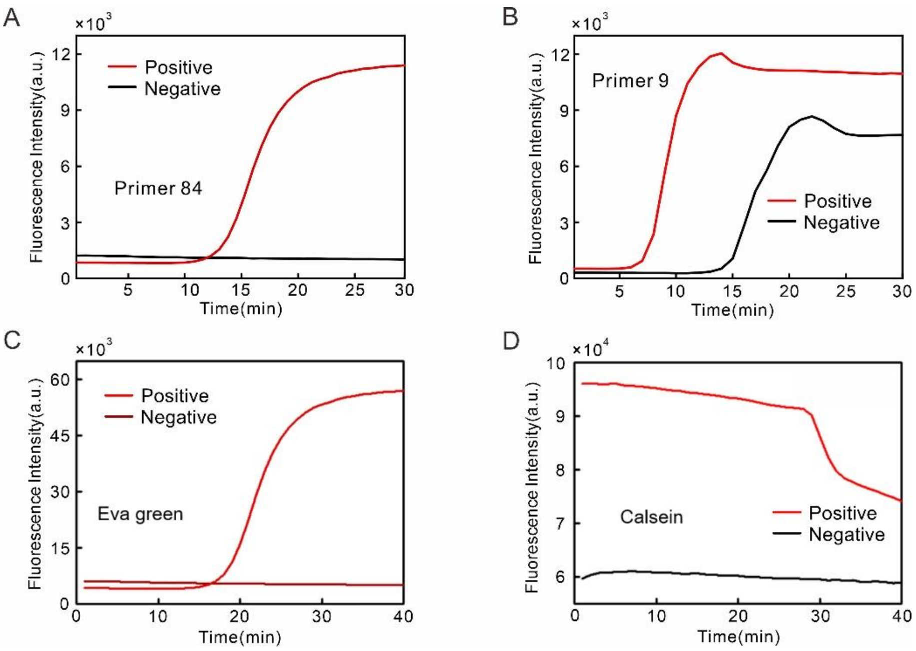

Figure S2: The selection of primers and dye. A) Amplification curves of SARS-CoV-2 gene using Primer 84; the result was prone to false positive. B) Amplification curves of gene using Primer 9, which could distinguish the positive sample from negative. C) Amplification curves of SARS-CoV-2 gene using EvaGreen as the dye for detection. The result of positive sample was clearly distinguished from negative sample. D) Amplification curves of the gene using Calsein as the dye. The curve of positive sample is difficult to distinguish the changes before and after the reaction, and the result was prone to false negative.

Table S3. Sensitivity of the LAMP assay for SARS-CoV-2 with the portable low-cost device.

Samples	Positive rate (positive samples/ total) (%)	Reaction time (min)
**1000 copies mL^{-1}**	7/10 (70 %)	15
	10/10 (100 %)	30
**500 copies mL^{-1}**	0/10	15
	2/10 (20 %)	30
**250 copies mL^{-1}**	0/10	15
	0/10	30

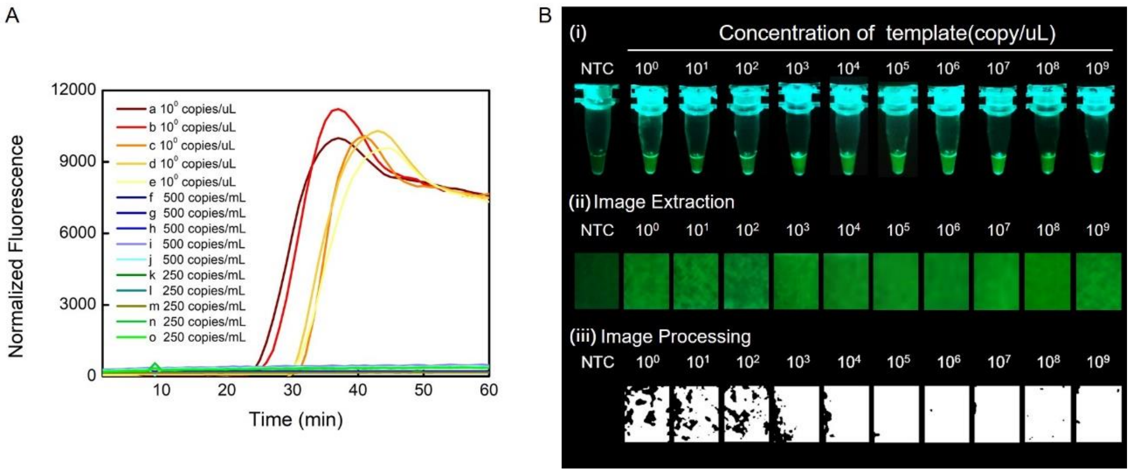

Figure S3: Amplification curves and image processing of samples with different template concentrations. A) Amplification curves of samples with different template concentrations from 250 copies/mL to 10^0 copies/μL; repeated tests of 10^0 copies/μL gene showed that the detection limit of this detection method could reach to 10^0 copies/μL. B) Image processing of samples with different template concentrations from 10^0 copies/μL to 10^9 copies/μL after 30 min amplification; white area in the processed image of sample with template concentration of 10^0 copies/μL is 74.5%.

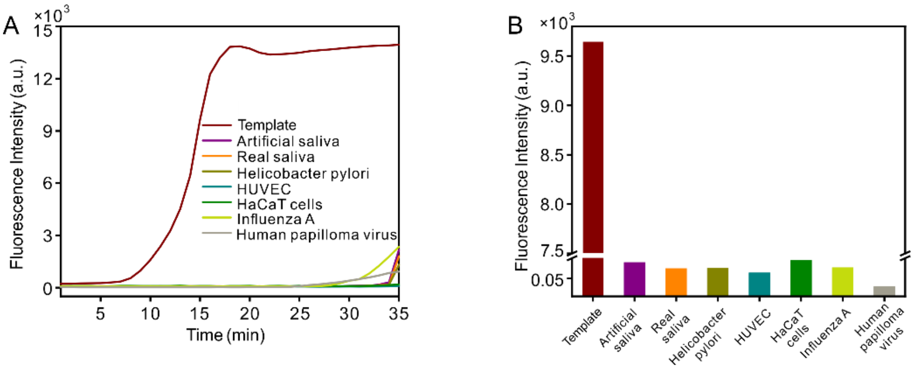

Figure S4: The specificity for detection of SARS-CoV-2 in the salivary environment using the portable low-cost device. A) The amplification curves of samples containing templates, artificial saliva, real saliva, Helicobacter pylori, HUVEC, HaCaT cells, influenza A, and human papilloma virus. B) Fluorescence intensity of samples after 15 minutes of reaction.

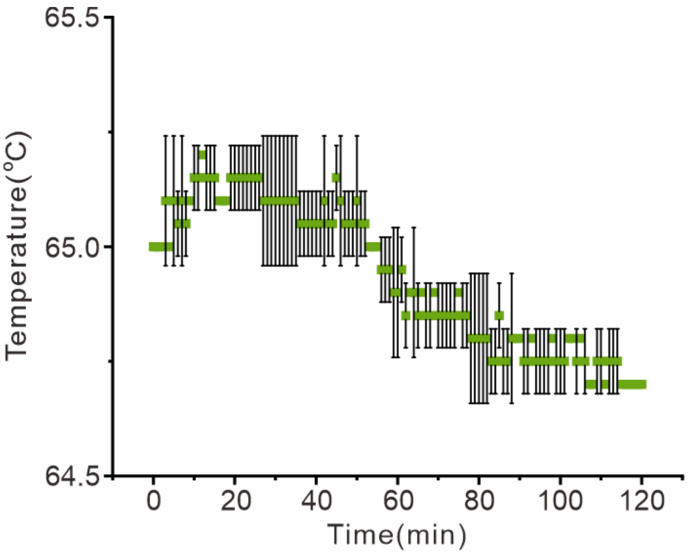

Figure S5: The temperature of water in the thermos could be kept around 65 ℃ with no more than 1 degree.

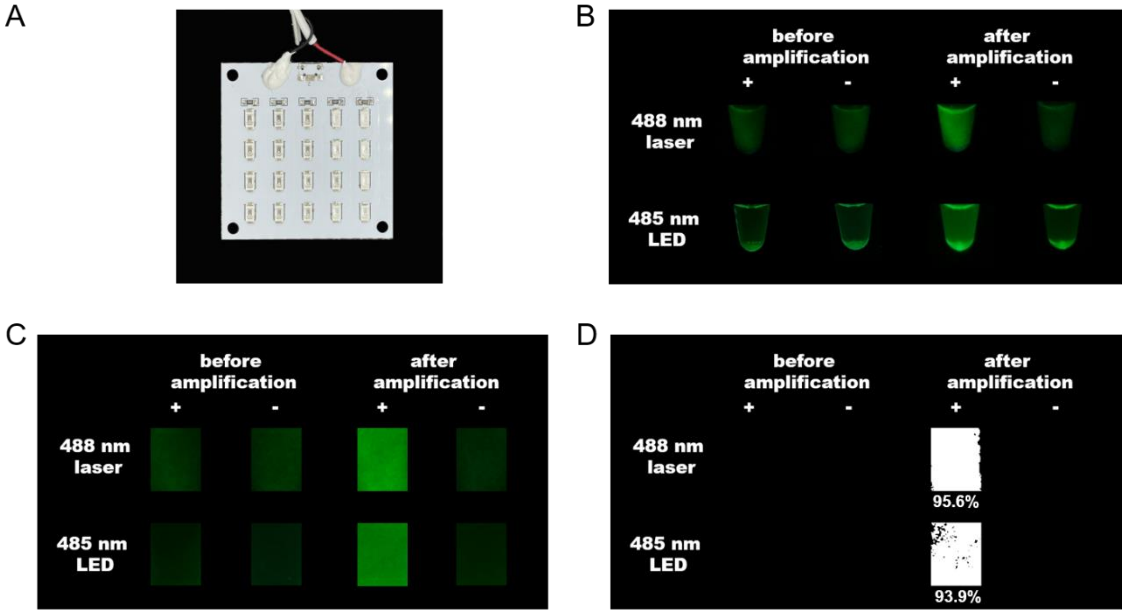

Figure S6: The fluorescence results under a 485 nm LED array and the 488 nm laser light sources are similar. A) Photo of a 485 nm LED array. B) Photos of samples under the excitation of a 488 nm laser and a 485 nm LED array. C) Extracted images of samples under the excitation of a 488 nm laser and a 485 nm LED array. D) Processed images of samples under the excitation of a 488 nm laser and a 485 nm LED array; the percentage of white area in the processed image of positive sample under the excitation of a 488 nm laser is 95.6%. Set the brightness value 66 as the threshold of the photo under LED excitation. The percentage of white area in the processed image of positive sample under the excitation of a 485 nm LED is 93.9%.

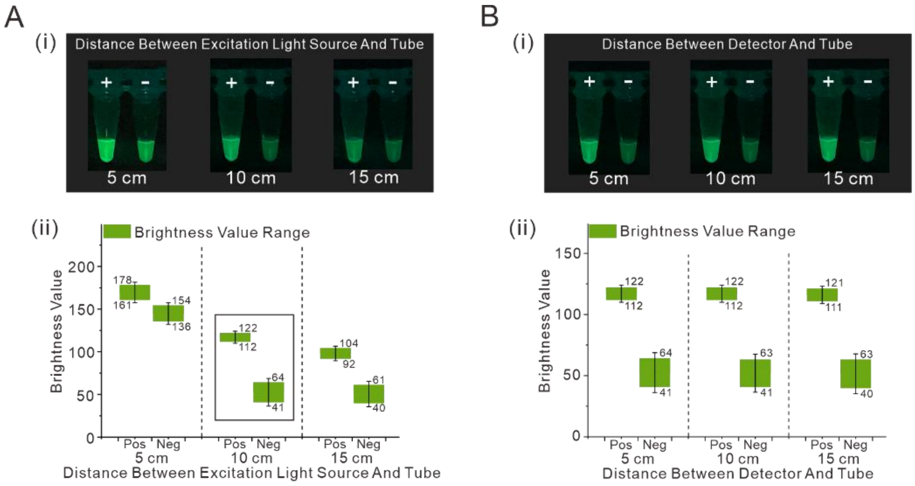

Figure S7. The brightness value of sample changed with the relative positions of the tube, excitation light source, and detector. A) The photo(i) and brightness value(ii) of sample with the distance of the tube and excitation light source changing. B) The photo(i) and brightness value(ii) of sample with the distance of the tube and detector changing.

Video S1: The assembly of the portable low-cost device for detecting SARS-CoV-2.

Table S4. The total time of the assay- from sample obtaining to results registration.

**Operation procedure**	**Time**
Obtain saliva sample	1min
Sample with a capillary	1 min
Adjust the water temperature to 65 ℃	2 min
Wait for LAMP	15 min
Take photos	1 min
Process images and record results	2 min
Total	22 min
